# Correction: Ventilation conditions during COVID-19 outbreaks in six California state carceral institutions

**DOI:** 10.1371/journal.pone.0330961

**Published:** 2025-08-22

**Authors:** The PLOS One Staff

## Notice of Republication

This article was republished on August 8, 2025, to correct an error in the fourth author’s name. The correct name is: David Sears. The correct citation is: Sklar R, Noth E, Kwan A, Sears D, Bertozzi S (2023) Ventilation conditions during COVID-19 outbreaks in six California state carceral institutions. PLoS One 18(11): e0293533. https://doi.org/10.1371/journal.pone.0293533

## Supporting information

S1 File Originally published, uncorrected article. (PDF)

S2 File Republished, corrected article. (PDF)
