# Supplementary material for: Correction: Ventilation conditions during COVID-19 outbreaks in six California state carceral institutions
Source: PLoS One. 2025 Aug 22;20(8):e0330961. doi: 10.1371/journal.pone.0330961 (PMC12373199; doi:10.1371/journal.pone.0330961)
Supplement: S1 File — (PDF) [file pone.0330961.s001.pdf]

PLOS ONE

RESEARCH ARTICLE

Ventilation conditions during COVID-19 outbreaks in six California state carceral institutions

Rachel Sklar[1]*, Elizabeth Noth[2], Ada Kwan[3], David Sear[4], Stefano Bertozzi[5,6]

1 Program on Reproductive Health and the Environment, University of California San Francisco, San Francisco, California, United States of America, **2** Division of Environmental Health Sciences, School of Public Health, University of California, Berkeley, Berkeley, CA, United States of America, **3** Division of Pulmonary and Critical Care Medicine, University of California, San Francisco, San Francisco, CA, United States of America, **4** Division of Infectious Diseases, Department of Medicine, School of Medicine, University of California, San Francisco, San Francisco, CA, United States of America, **5** Division of Health Policy and Management, School of Public Health, University of California, Berkeley, Berkeley, California, United States of America, **6** School of Public Health, University of Washington, Seattle, Washington, USA and Instituto Nacional de Salud Pública, Cuernavaca, Mexico

* rachel.sklar@ucsf.edu

Check for updates

OPEN ACCESS

Citation: Sklar R, Noth E, Kwan A, Sear D, Bertozzi S (2023) Ventilation conditions during COVID-19 outbreaks in six California state carceral institutions. PLoS ONE 18(11): e0293533. https://doi.org/10.1371/journal.pone.0293533

Editor: Ahmed Mancy Mosa, Al Mansour University College-Baghdad-Iraq, IRAQ

Received: March 3, 2023

Accepted: September 23, 2023

Published: November 7, 2023

Copyright: © 2023 Sklar et al. This is an open access article distributed under the terms of the Creative Commons Attribution License, which permits unrestricted use, distribution, and reproduction in any medium, provided the original author and source are credited.

Data Availability Statement: The data can be found in a supplementary csv file that has been submitted as the S1 File.

Funding: The results of this manuscript are associated with a project to better understand transmission and mitigation factors associated with COVID-19 in California's state prisons. The project was funded by the California Prison Receivership Office that oversees delivery of health care services under the federal courts in California's state prison system. The funders had no role in the study design, data collection and

Abstract

Residents of carceral facilities are exposed to poor ventilation conditions which leads to the spread of communicable diseases such as COVID-19. Indoor ventilation conditions are rarely studied within carceral settings and there remains limited capacity to develop solutions to address the impact of poor ventilation on the health of people who are incarcerated. In this study, we empirically measured ventilation rates within housing units of six adult prisons in the California Department of Corrections and Rehabilitation (CDCR) and compare the measured ventilation rates to recommended standards issued by the World Health Organization (WHO). Findings from the empirical assessment include lower ventilation rates than the recommended ventilation standards with particularly low ventilation during winter months when heating systems were in use. Inadvertent airflows from spaces housing potentially infected individuals to shared common spaces was also observed. The methodology used for this work can be leveraged for routine ventilation monitoring, pandemic preparedness, and disaster response.

Introduction

During the spring and summer of 2020, COVID-19 case rates in prison were more than five times the rate of the non-incarcerated U.S. population [1]. In other congregate living facilities, the importance of indoor air quality and ventilation has been widely demonstrated [2, 3]. To our knowledge, no studies have described the means by which ventilation conditions in carceral settings may impact COVID-19 transmission. Addressing this knowledge gap is particularly important given the aging infrastructure and overcrowding in many prisons, as well as the increased vulnerability to complications from infectious diseases or respiratory health issues among incarcerated populations and carceral employees [4, 5].

analysis, or decision to prepare and publish the manuscript. The California Prison Receivership Office reviewed the manuscript before submission with no changes requested to the content of the manuscript. All authors were funded by CDCR to carry out outbreak investigation efforts within the California Department of Corrections and Rehabilitation including but not limited to the work presented in this manuscript.

Competing interests: The authors have declared that no competing interests exist.

Respiratory droplets and aerosols containing the SARS-CoV-2 virus exhaled by infected individuals may remain suspended in air for significant periods of time [6].Ventilation works to prevent transmission of the virus indoors by removing air containing viral particles and replacing it with clean air. Poorly ventilated indoor spaces pose a particularly high risk for individuals living or working in high occupancy settings where many individuals may be exposed to air containing infectious viral particles [7].

Carceral facilities in the U.S. tend to be of older construction and there is minimal data on the existence and performance of their ventilation systems. In California, the average age of the 35 California Department of Corrections and Rehabilitation's (CDCR's) state correctional facilities exceeds 45 years with approximately 35 percent of the facilities exceeding 50 years of age including two facilities with buildings dating back to the 1800s [8]. Thus, the lack of data on ventilation systems within carceral settings hinders the development of infection control strategies. This knowledge gap also prevents ventilation guidelines from being a part of pandemic preparedness plans and overall ventilation for maintining health in the context of other disasters (e.g. wildfires) in carceral settings.

In this paper we focus on characterizing the ventilation conditions within carceral settings by measuring ventilation rates and direction of airflow in housing units within CDCR. Our findings—particularly when compared to WHO standards for ventilation in healthcare and quarantine facilities—can inform recommendations about pandemic preparedness in the prison context.

Materials and methods

Study sample

We conducted quantitative assessments of ventilation conditions in housing units of six prisons within the CDCR state prison system during COVID-19 outbreaks. At the time of our sampling, there were 35 operational adult prison facilities within CDCR. All ventilation sampling was conducted between December 2020 and June 2021.

Three main types of housing units were sampled: 1) celled housing units with solid doors, 2) celled housing units with open-grate doors, and 3) dormitory units. The celled housing units were buildings in which two to five stories of one to two person cells were arranged around a central atrium, also known as a dayroom. The dormitory units were single or double story buildings containing bunk beds in an open space.

Ventilation estimation

To estimate ventilation rates in one- and two-person cells, a tracer gas concentration decay method was used in cells that were vacant [9]. Cells with solid doors and cells with open-grate doors, which allowed for open exchange of air between the cell and the outside atrium were measured. Because dormitories could not be vacated, CO_2 was measured as a natural tracer gas to assess ventilation [10]. A TSI9535 Velocicalc (TSI, Shoreview, MN) was used to measure the CO_2 concentrations in both cells and dorms. To estimate per-person ventilation rates, the air exchange rates were converted to cubic feet per minute (CFM) and divided by the number of occupants in the room.

Static pressure of cells

The direction of airflow between cells and common spaces within housing units was determined by sampling static pressure in cells relative to either the hallway or dayroom. The

pressure differential inside and outside the cells was assessed using the VelociCalc and pitot probe attachment under the door of the cell.

Results

Ventilation rate

The median measured (or estimated) ventilation rates in cells and dormitories ranged from 18.1 CFM/person to 69.9 CFM/person (Table 1). All of the cells measured had ventilation rates less than the WHO recommended minimum of 60 L/S, or 127 CFM/person for healthcare and quarantine facilities [11].

Ventilation rates in summer versus winter

Three facilities were visited during both summer (June 2021) and winter (December 2020/January 2021). The estimated ventilation rate per person was lower in the winter than in the summer for cells and dorms across all facilities with the exception of facility 2 in which the ventilation rates did not differ significantly between the two seasons. It is important to note that for the purposes of energy efficiency and cost savings in the winter, heating systems were operated in recirculation mode. This means the same heated and potentially virus laden air is recirculated through the building as opposed to ventilation and dilution of potential viral contaminants with fresh outdoor air. Also during the winter, doors to the housing facilities we visited were closed to conserve heat and limit fresh air entry to the housing units.

Ventilation rates in cells with open-grate doors versus solid doors

At facility 3 the cells with the open-grate door types had significantly lower air exchange than those in units with closed-door cells in both winter and summer. This is in contrast to facility 4 where closed-door cells had lower air exchange than open grate door types, highlighting heterogeneity in ventilation systems across the CDCR system.

Static pressure of cells

The majority of solid-door cells sampled were under positive pressure, meaning airflow was from the cell to the common areas outside of the cell such as hallways or dayrooms. The

Table 1. Ventilation characteristics of six CDCR facilities, 2020–2021.

			Cells					Dorms			
Facility #	Cell Door Type	Season	N	Median CFM pp	Min CFM pp	Max CFM pp	% Under Positive Pressure	N	Median CFM pp	Min CFM pp	Max CFM pp
1	solid	winter	7	43.2	23.5	61.3	100%	3	70.5	46.0	116.5
1	solid	summer	13	66.2	30.9	157.8	62%		NA		
2	solid	winter	4	46.1	17.1	124.8	NA	1	36.0	36.0	36.0
2	solid	summer	6	43.4	22.7	80.9	NA	2	59.3	14.7	104.0
3	open grate	winter	6	18.1	11.8	89.7	NA	0	NA		
3	open grate	summer	24	51.8	29.4	165.2	NA	0	NA		
3	solid	winter	1	69.9	69.9	69.9	100%	0	NA		
3	solid	summer	1	105.8	105.8	105.8	NA	0	NA		
4	open grate	summer	8	54.0	23.7	144.5	NA	0	NA		
4	solid	summer	1	27.5	27.5	27.5	100%	0	NA		
5	solid	summer	10	63.2	27.4	131.6	100%	0	NA		
6	solid	summer	4	45.3	27.1	46.0	100%	4	75.1	73.2	76.9

https://doi.org/10.1371/journal.pone.0293533.t001

exception was in facility one in which inconsistenincies in the pressure and airflow direction were measured within solid-door cells of the same building. No pressure gradient was observed between open-grate cells and common spaces.

Discussion

Our study describes ventilation conditions that were measured at CDCR facilities during the COVID-19 pandemic. Notable findings include low per-person ventilation rates compared to WHO standards, worsened ventilation rates in winter months, and positive static pressure in cells (potentially moving infectious air from cells to common areas), all of which may heighten the risk of COVID-19 transmission among residents and staff. None of the housing units we measured met the minimum 127 CFM/person ventilation rate guideline set by WHO for indoor ventilation in health care and quarantine facilities, and which has been estimated to decrease airborne disease transmission by up to 38% in other studies [11, 12]. The median ventilation rates measured exceeded minimum ventilation rate recommendations for correctional facilities in non-pandemic scenarios. These standards were initially designed to control body odor and we argue that carceral facilities need to increase ventilation rates in the event of a pandemic such that the ventilation function is geared for infection control [13, 14].

The lower air exchange rates observed in the same housing units in the winter versus the summer suggest the role of heating and recirculation systems, as well as the tendency to keep building doors closed in the winter, in reducing overall ventilation rates. During our initial winter visits in December 2020, as much as 90% of the air within housing units was recirculated heated air and the doors were closed to all housing units that we visited. Consequentially, transmission risk within the housing units we sampled could have been significantly higher during the winter months. Following the release of our results, CDCR policy subsequently dramatically reduced recirculation as the risk became apparent. To avoid increased transmission during winter months, facilities should avoid recirculating heated air to the extent possible and instead rely on as much outdoor air as possible. In settings where air conditioning (as opposed to swamp coolers) is used in summer months, the same would apply. Where it is not feasible to use outdoor air, effective filtration is essential [15].

The positive pressure observed in most cells sampled indicates the airflow from potentially infected cells to common areas. Despite isolating individuals from each other in single-person cells within isolation and quarantine units, transmission ensued. In a prison pandemic scenario, isolation and quarantine units in particular should be under negative, rather than positive, pressure. A cell under negative pressure would have a lower air pressure inside the cell compared to the outside common space and ensure that fresh air flows into the room while any harmful virus laden air located inside the room is removed with exhaust systems. Negative pressure rooms are commonly used within healthcare settings to avoid the spread of disease and a core part of the WHO ventilation recommendation in the context of COVID-19. Alternatively, ultraviolet irradiation could be used in common spaces to disinfect air that is on the return path from individual cells [16].

The heterogeneity in the pressure and airflow direction from cells within the same building in facility 1 can be attributed to the poor performance of supply and return vents in individual cells or the practice of residents covering vents with cardboard and cloths to block the entrance of cold air. These factors may also explain why ventilation rates were higher in the solid cells sampled in facility 3, but lower in facility 4. Regular rebalancing exercises can ensure that vents within the system are performing to specification, avoiding inadvertent airflow from areas holding infected individuals to areas with uninfected individuals.

The combination of low ventilation rates and cells under positive pressure suggest the dangers of housing people with unknown infection status in the same building. Despite isolation that is established through individual cells with solid doors, positive pressure from cells to common areas can promote the movement of virus-laden air to areas with uninfected individuals. In cell blocks with open-grate cells, no pressure gradient was observed suggesting a bidirectional flow of air between cells and common areas and the potential for infectious agents generated in any cell to diffuse freely throughout the building.

Our findings on low ventilation conditions within prison housing units have implications for other health outcomes in incarcerated populations and prison staff. Improving ventilation rates and filtration in housing units can reduce the transmission of other airborne diseases such as tuberculosis that are highly prevalent in incarcerated populations [17, 18]. Poor ventilation not only leads to the transmission of infectious diseases, it can also exacerbate the exposure of incarcerated populations to extreme heat [19]. In California, prisons are built in areas that are more prone to extreme heat and wildfire events that are growing in their frequency and intensity. Ventilation improvements are a basic step in mitigating the health risks that these growing environmental threats pose on populations. The methods that we present in this paper can be used to characterize ventilation systems and prioritize improvements.

It its important to note that some practices aimed at controlling COVID-19 may work to exacerbate exposure to wildfire smoke. For example, minimizing air recirculation and introducing as much fresh outdoor air as possible is a strategy for decreasing COVID-19 transmission risk. In contrast, to minimize smoke exposure during a fire event, the use of recirculation mode on the air conditioning unit should be maximized to prevent outdoor smoke from entering the indoor space [20]. Further work is needed to develop specific guidance for facilities that may be balancing the need to simultaneously control for wildfire smoke exposure and COVID-19 transmission.

Conclusions

Despite the toll that COVID-19 has had on people who are incarcerated, there has been little understanding of successful mechanisms for reducing transmission in these facilities. We conclude that ventilation improvements should be a central component of emergency respiratory pandemic preparedness and response plans. Given the location of prisons in wildfire prone areas, properly functioning ventilation and air filtration systems are a minimum requirement in reducing disproportionate exposures to smoke for incarcerated populations. Ventilation assessments such as the ones that were conducted here can be used on an ongoing basis to identify high-risk areas and monitor the impact of mitigation measures designed to improve ventilation. The methods that we use here are relevant for incarcerated populations in California as well as other states where overcrowding, poorly maintained ventilation systems, and susceptible residents are subjected to disproportionate exposure to health risks and disease.

Supporting information

S1 File. This is ventilation data collected for six facilities.
(CSV)

Acknowledgments

We offer our sincere gratitude to those who helped us conduct this research, particularly the residents and staff of California prisons, as well as those who have been working to respond to COVID-19 within prisons and jails across the country.

Author Contributions

Conceptualization: Rachel Sklar, Elizabeth Noth, Ada Kwan, David Sear, Stefano Bertozzi.

Data curation: Rachel Sklar.

Formal analysis: Rachel Sklar.

Investigation: Rachel Sklar, Elizabeth Noth.

Methodology: Elizabeth Noth.

Project administration: Rachel Sklar.

Supervision: Stefano Bertozzi.

Writing – original draft: Rachel Sklar.

Writing – review & editing: Elizabeth Noth, Ada Kwan, David Sear, Stefano Bertozzi.

References

1. Saloner B, Parish K, Ward JA, DiLaura G, Dolovich S. COVID-19 Cases and Deaths in Federal and State Prisons. JAMA. 2020; 324: 602–603. https://doi.org/10.1001/jama.2020.12528 PMID: 32639537
2. Wang CC, Prather KA, Sznitman J, Jimenez JL, Lakdawala SS, Tufekci Z, et al. Airborne transmission of respiratory viruses. Science. 2021; 373: eabd9149. https://doi.org/10.1126/science.abd9149 PMID: 34446582
3. Morawska L, Tang JW, Bahnfleth W, Bluyssen PM, Boerstra A, Buonanno G, et al. How can airborne transmission of COVID-19 indoors be minimised? Environ Int. 2020; 142: 105832. https://doi.org/10.1016/j.envint.2020.105832 PMID: 32521345
4. Binswanger IA, Krueger PM, Steiner JF. Prevalence of chronic medical conditions among jail and prison inmates in the USA compared with the general population. Journal of Epidemiology & Community Health. 2009; 63: 912–919. https://doi.org/10.1136/jech.2009.090662 PMID: 19648129
5. Nowotny KM, Seide K, Brinkley-Rubinstein L. Risk of COVID-19 infection among prison staff in the United States. BMC Public Health. 2021; 21: 1036. https://doi.org/10.1186/s12889-021-11077-0 PMID: 34078350
6. van Doremalen N, Bushmaker T, Morris DH, Holbrook MG, Gamble A, Williamson BN, et al. Aerosol and Surface Stability of SARS-CoV-2 as Compared with SARS-CoV-1. N Engl J Med. 2020; NEJMc2004973. https://doi.org/10.1056/NEJMc2004973 PMID: 32182409
7. Buonanno G, Stabile L, Morawska L. Estimation of airborne viral emission: Quanta emission rate of SARS-CoV-2 for infection risk assessment. Environment International. 2020; 141: 105794. https://doi.org/10.1016/j.envint.2020.105794 PMID: 32416374
8. Macomber J, Barretto J, Lief C. Master Plan Annual Report.
9. ASTM. E741-11(2017). Standard Test Method for Determining Air Change in a Single Zone by Means of a Tracer Gas Dilution. 2017. [cited 17 Aug 2022]. Available: https://www.astm.org/e0741-11r17.html
10. ASTM. D6245-07. Standard Guide for Using Indoor Carbon Dioxide Concentrations to Evaluate Indoor Air Quality and Ventilation. 2007. [cited 17 Aug 2022]. Available: https://www.astm.org/d6245-07.html
11. World Health Organization. Roadmap to improve and ensure good indoor ventilation in the context of COVID-19. Geneva: World Health Organization; 2021. Available: https://www.who.int/publications-detail-redirect/9789240021280
12. Andrews JR, Yeckel CW, Sgarbi RVE, Croda J, Urrego J, Paião DSG, et al. The Impact of Ventilation and Early Diagnosis on Tuberculosis Transmission in Brazilian Prisons. The American Journal of Tropical Medicine and Hygiene. 2015; 93: 739–746. https://doi.org/10.4269/ajtmh.15-0166 PMID: 26195459
13. ANSI/ASHRAE Standard 62.1–2019 Ventilation for Acceptable Indoor Air Quality. ASHRAE; 2019. Available: https://www.ashrae.org/technical-resources/bookstore/standards-62-1-62-2
14. Persily A. Challenges in developing ventilation and indoor air quality standards: The story of ASHRAE Standard 62. Building and Environment. 2015; 91: 61–69. https://doi.org/10.1016/j.buildenv.2015.02.026 PMID: 31274954
15. Azimi P, Stephens B. HVAC filtration for controlling infectious airborne disease transmission in indoor environments: Predicting risk reductions and operational costs. Build Environ. 2013; 70: 150–160. https://doi.org/10.1016/j.buildenv.2013.08.025 PMID: 32288024

16. Walker CM, Ko G. Effect of Ultraviolet Germicidal Irradiation on Viral Aerosols. Environ Sci Technol. 2007; 41: 5460–5465. https://doi.org/10.1021/es070056u PMID: 17822117

17. Baussano I, Williams BG, Nunn P, Beggiato M, Fedeli U, Scano F. Tuberculosis Incidence in Prisons: A Systematic Review. PLOS Medicine. 2010; 7: e1000381. https://doi.org/10.1371/journal.pmed.1000381 PMID: 21203587

18. Dara M, Acosta CD, Melchers NVSV, Al-Darraji HAA, Chorgoliani D, Reyes H, et al. Tuberculosis control in prisons: current situation and research gaps. International Journal of Infectious Diseases. 2015; 32: 111–117. https://doi.org/10.1016/j.ijid.2014.12.029 PMID: 25809766

19. Skarha J, Peterson M, Rich JD, Dosa D. An Overlooked Crisis: Extreme Temperature Exposures in Incarceration Settings. Am J Public Health. 2020; 110: S41–S42. https://doi.org/10.2105/AJPH.2019.305453 PMID: 31967879

20. California Air Resources Board. Smoke Ready California: Protecting Yourself from Wildfire Smoke. Sacramento: California Air Resources Board. [cited 20 Jul 2023]. Available: https://ww2.arb.ca.gov/protecting-yourself-wildfire-smoke#:~:text=Stay%20Indoors%20and%20Keep%20Indoor,with%20windows%20and%20doors%20closed.
